# Gender and sexual identity-based inequalities in adolescent wellbeing: findings from the #BeeWell Study

**DOI:** 10.1186/s12889-023-16992-y

**Published:** 2023-11-09

**Authors:** Jose Marquez, Neil Humphrey, Louise Black, Megan Cutts, Devi Khanna

**Affiliations:** Manchester Institute of Education, University of Manchester, Manchester, M13 9PL UK

**Keywords:** Inequalities, Adolescence, Wellbeing, Gender identity, Sexual identity

## Abstract

**Background:**

Gender and sexual minority adolescents experience greater symptoms of psychological distress than their peers, but little is known about broader aspects of their wellbeing. This study examines wellbeing inequalities relating to gender and sexual identity among adolescents from Greater Manchester in the United Kingdom.

**Method:**

37,978 adolescents (aged 12–15, attending 165 secondary schools) completed surveys of life satisfaction, positive and negative affect (hedonic framework); autonomy, self-esteem, optimism, and positive relationships (eudaimonic framework); and, symptoms of distress and mental wellbeing (complete state framework). Structural correlated factors models were used to assess gender and sexual identity wellbeing inequalities.

**Results:**

The magnitude of wellbeing inequalities pertaining to gender and sexual identity were routinely substantially greater than those concerning other characteristics (e.g., socio-economic disadvantage). Gender identity wellbeing inequalities followed a consistent pattern, with the largest disparities evident between gender diverse adolescents and boys. Sexual identity wellbeing inequalities also followed a consistent pattern, with the largest disparities evident between sexual minority youth (both gay/lesbian and bi/pansexual) and their heterosexual peers. Finally, variation was evident across wellbeing domains. For example, observed gender identity (boys vs. girls) and sexual identity (heterosexual vs. sexual minority) disparities were substantially greater for symptoms of distress than for mental wellbeing in the complete state model.

**Conclusions:**

LGBTQ + adolescents experience lower wellbeing than their peers, and this is evident across a range of wellbeing domains. Accordingly, there is an urgent need for the prioritisation of improved prevention and intervention efforts that can better meet the needs of gender diverse and sexual minority youth, and future research should be conducted to improve understanding of the mechanisms underpinning the wellbeing inequalities observed.

**Supplementary Information:**

The online version contains supplementary material available at 10.1186/s12889-023-16992-y.

## Introduction

Inequalities are systematic, avoidable and unfair differences in outcomes between different populations or groups [1]. In this paper we focus on inequalities in adolescent wellbeing across gender and sexual identity. While much is known about this topic in relation to other characteristics such as age, socio-economic status, ethnicity and binary categorisations of gender and sex [2–4], our understanding of the nature and magnitude of wellbeing disparities between gender/sexual minority youth and their peers remains limited, especially in early adolescence [5]. This is an important research problem to address given that this is the developmental stage when young people develop their gender and sexuality identities [6], and wellbeing in this period is known to impact outcomes across the lifespan [7].

### What do we mean by wellbeing?

A critical starting point is the lack of a universally accepted definition of wellbeing in the contemporary literature [8–10]. It is often conjoined or used interchangeably with ‘mental health’ [11, 12], a term that similarly eludes precise definition [13]. Being precise about the particular domains and theory that are considered in a given wellbeing study is therefore critical to further our understanding. A number of conceptual frameworks of wellbeing have been developed. The *hedonic* approach (also referred to as subjective wellbeing) operationalises wellbeing as comprising affective (positive and negative affect) and cognitive (life satisfaction) components, and emphasizes ‘feeling good’. The *eudaimonic* approach (also referred to as psychological wellbeing) can include autonomy, environmental mastery, optimism, personal growth, positive relations with others, purpose in life, and self-acceptance, and emphasizes ‘flourishing’ [14]. Finally, the *complete state* approach (also known as the dual factor/continuua or complete mental health model) focuses on the balance between mental wellbeing and symptoms of distress, emphasizing the presence of positive states and absence of symptoms [15]. Research that draws upon these wellbeing frameworks has proliferated in recent years, though to our knowledge, the current study is the first to apply them systematically to advance understanding of inequalities in adolescence.

### Wellbeing in adolescence: what do we know?

Adolescence, which begins around age 10, is marked by significant hormonal, physical, neurobiological, psychological, social, and environmental changes [16], and is a period that confers significant vulnerability in certain aspects of wellbeing [17]. Wellbeing starts to decline in the transition from childhood to adolescence [2]. Adolescence is also the time when the prevalence of significant mental health difficulties increases markedly [18], and the majority of lifetime cases of mental ill health manifest by the age of 24, with a peak age of onset of 14.5 years [19].

Current national data indicates that 17.7% of 11-to-16 year-olds in England have a probable mental health disorder [20]. While concerning, these figures do however make clear the need to also capture data on wellbeing indicators in population mental health research [21]. Aside from the alignment with the theoretical frameworks outlined above, gathering data on both symptoms of distress *and* broader wellbeing likely captures greater variability than is possible with a solely symptom-driven approach, because while most individuals are asymptomatic, there remains considerable variability in life satisfaction and other such indicators [22]. Furthermore, concerning trends have been observed in relation to these data in multiple countries [23–25], including a clear decline in adolescent life satisfaction in the UK in the decade from 2009 to 2019 [24], with the four UK nations leading a worldwide decline between 2015 and 2018 [25]. Given that wellbeing in adolescence is predictive of adult wellbeing, labour market/socioeconomic, physical health/health behaviour, and relational outcomes [7], it is vital that we understand more about what is clearly a critical period.

### What do we mean by gender and sexual identity?

Similar to wellbeing, gender and sexuality concepts and terms can be challenging to define, are not universally agreed upon, often are used interchangeably, and reflect a range of meanings. Accordingly, we clarify our intended usage/meaning of them for the reader. First, we distinguish between sex (classification as male, female, or intersex, on the basis of biological/chromosomal factors) and gender (the socially and culturally constructed norms, behaviours, characteristics and roles associated with being male, female, or somewhere outside of this basic binary) [26, 27]. When referring to gender identity, we mean the feeling or sense of oneself as being female, male, a blend of both, or neither [26, 28]. Thus, sex and gender interact but are different [28]. To aid clarity and precision, throughout the article we use male and female when referring to sex, and boy, girl, and terms reflecting the non-binary nature of gender (gender diverse) when referring to gender identity. Following Ashley [29], we also use the term gender modality, which refers to how an individual’s gender identity stands in relation to their assignment as male or female at birth, making two classifications: cisgender (where these correspond) and transgender (where these do not correspond). Finally, we make reference to sexual identity, by which we mean how an individual thinks of themselves in terms of their emotional, romantic and/or sexual attraction to other people, often categorised in relation to the gender/sex classifications noted above [30]. At the outset, we note that categorising adolescents in terms of their gender and sexuality is challenging as this is a life stage in which identities are developing [6], and we also acknowledge that there are other valid ways to categorise young people according to their gender and sexuality that can provide useful research insights [31, 32].

### Wellbeing among gender and sexual minority adolescents: what do we know?

Minority stress theory [33] suggests adolescents who are lesbian, gay, bisexual, transgender or questioning (LGBTQ+) are likely to experience lower wellbeing than their peers. It predicts adverse health outcomes among gender and sexual minority youth as a result of excess stress emanating from experiences of prejudice, discrimination, sexual identity concealment, expectations of rejection, and internalised stigma [34–36]. This may be compounded by punitive and traumatic reactions from parents and caregivers in response to their children’s identity [37].

There is evidence of marked inequalities in relation to gender and sexual identity for certain adolescent wellbeing indicators, specifically those pertaining to mental health difficulties. For example, a systematic review by Plöderl and Tremblay [5] revealed elevated risks for depression, anxiety, suicide attempts or suicides among sexual minority adolescents (e.g., those who are attracted to the same or both sexes). In a recent illustrative case, Amos et al’s [38] national cohort study in the UK found that sexual minority adolescents were more than five times more likely than their heterosexual peers to experience high depressive symptoms. Additionally, a systematic review by Connolly et al. [39] revealed significantly higher rates of depression, suicidality, self-harm and eating disorders among transgender youth when compared to their cisgender peers.

In considering inequalities pertaining to gender identity, it is important not to overlook those between boys and girls. A major international analysis (> 500,000 adolescents, > 70 countries) by Campbell et al. [4] found consistent evidence of substantial inequalities in relation to indicators of symptoms of distress, life satisfaction, hedonia and eudaimonia, with girls reporting worse outcomes than boys in all cases. There are multiple sociocultural, psychological, and biological factors underpinning such findings, which likely operate in parallel to create chronic stress for girls [40]. Among these factors, gender norms (e.g., the implicit ‘rules’ that guide which attributes and behaviours are valued and accepted for boys and girls) may be particularly important, as those adopted in adolescence arguably reflect and reinforce inequitable hierarchies, with concurrent and consequent negative effects on girls’ health and wellbeing theorised to operate via differential risk factor exposures, health-related behaviours, and access to care [41–43].

### The current study

Building on the above, we sought to examine wellbeing inequalities relating to gender and sexual identity by leveraging analysis of a unique contemporary adolescent dataset from a project known as #BeeWell, which we describe in more detail in the following section (see Method). We aim to provide a deeper, more rigorous, and nuanced analysis of the nature and scale of adolescent wellbeing inequalities than has been possible to date, by moving beyond those that pertain directly to symptoms of distress (which have dominated the literature considering gender and sexual identity [44]). Moreover, noting a critical concern expressed by Green et al. [45], we analyse data on sexual identity *and* gender identity (with there having being large gaps in relation to the latter in the extant literature), permitting more granular, segmented analyses than are currently evident. Furthermore, noting that a growing number of young people report gender diverse identities that differ from social and cultural norms relating to rigid, binary constructs (e.g., boys vs. girls [46]), we use a contemporary dataset in which this is more accurately reflected. Finally, given that the majority of studies of this kind are US-based and typically focus on the latter stages of adolescence (e.g., only 15% of studies in the aforementioned review by Plöderl and Tremblay [5] were based in Europe, and most studies in the adolescent tranche included participants aged 16+), we aim to update and extend the evidence base to England, in relation to one of the most neglected stages of development: early adolescence [47].

In sum, the aim of the current study is to establish the nature and magnitude of early adolescent wellbeing inequalities, with a particular focus on gender and sexual identity. The research questions (RQs) driving the study are as follows.

- RQ1: What is the nature and magnitude of adolescent wellbeing inequalities pertaining to gender and sexual identity?
- RQ2: Are there any consistent patterns pertaining to the nature and magnitude of gender identity wellbeing inequalities?
- RQ3: Are there any consistent patterns pertaining to the nature and magnitude of sexual identity wellbeing inequalities?
- RQ4: Does the nature and magnitude of adolescent sexual identity wellbeing inequalities vary by how gender identity is categorised (i.e., by gender identity vs. gender modality)?
- RQ5: Do adolescent gender and sexual identity wellbeing inequalities vary across frameworks and domains (e.g., hedonic, eudaimonic, complete state)?

## Method

### Design

#BeeWell uses a hybrid population cohort study design [48], comprising two main elements: (i) a longitudinal study in which participants are tracked with annual data points from age 12–15 (e.g. from Year 8 to Year 9 to Year 10 of secondary school; Sample 1); and, (ii) a serial cross-sectional study comprising annual data points for participants aged 14–15 (e.g. those in Year 10 of secondary school at a given data point; Sample 2). Our analysis draws on the first annual wave of #BeeWell data collection (Autumn 2021), combining data from Samples 1 and 2. Further information about the #BeeWell study, dataset, and publications can be found on the project website (www.beewellprogramme.org).

### Participants

165 secondary schools across the 10 Local Authorities in the Greater Manchester city-region are represented in the study (63% of all secondary education settings in Greater Manchester in 2021). A total of N = 37, 978 adolescents completed surveys. This exceptional response rate, which represents more than 50% of *all* young people in eligible year groups (i.e. Years 8 and 10) living in Greater Manchester in 2021, was made possible by the outstanding support offered by project partner organisations, participating schools, and their pupils. In relation to the latter, the extensive consultation process undertaken during the #BeeWell survey design phase should also be noted (outlined below in the *Measures* subsection), given the considerable effort undertaken to produce a survey that was relevant, meaningful, and accessible to young people. More details about the study sample are presented in Table 1.

**Table 1:** Sample Composition and Equivalent National Data

Characteristic	Sample Composition	National Average
Sex	50.8% male, 49.2% female	50.3% male, 49.7% female
Gender identity	41.7% boy (inc trans boy), 40.0% girl (inc trans girl), 2.4% non-binary, 2.8% describe myself in another way, 5.3% prefer not to say, 7.9% missing	N/A
Sexual identity	67.5% heterosexual/straight, 2.7% gay/lesbian, 7.7% bi/pansexual, 3.7% describe myself in another way, 9.1% prefer not to say, 9.4% missing	N/A
Gender modality	79.7% cisgender, 7.1% transgender, 13.2% missing	N/A
Ethnicity	2.3% Any Other Ethnic Group. 18.1% Asian, 5.5% Black, 0.8% Chinese, 5.9% Mixed, 1.9% Unclassified, 65.6% White	2.2% Any Other Ethnic Group. 12.0% Asian, 6.2% Black, 0.5% Chinese, 6.3% Mixed, 2.0% Unclassified, 70.8% White
Special educational needs	85.8% No, 14.2% Yes	85.9% No, 14.1% Yes
Free school meal eligibility (in the last 6 years)	74.6% No, 25.4% Yes	N/A^a^
Year group	51.5% Year 8, 48.5% Year 10	50.9% Year 8, 49.1% Year 10

*Note*. National data dervied from Explore Education Statistics online tool [79]

^a^National data are not available for free school meal eligibility in the last 6 years (known as EverFSM6). National data forcurrent free school meal eligibility indicate that 20.9% of pupils aged 11–16 are eligible

### Measures

#### Gender and sexual identity

Items pertaining to gender and sexual identity were developed in consultation with #BeeWell Young Peer Reviewers and national LGBTQ + organisations. In relation to gender identity, participants were asked if they were a girl (including trans girl); boy (including trans boy); non-binary; describe themselves in another way; or, prefer not to say. A gender modality variable (transgender/cisgender) was created by examining the correspondence between self-reported gender identity and linked administrative data on sex (see Covariates section below). Sexual identity was probed by asking participants what best described them, from the following options: Bi/pansexual; gay/lesbian; heterosexual/straight; describe themselves in another way; or, prefer not to say.

#### Wellbeing

A range of wellbeing indicators and measures were available, spanning the aforementioned major theoretical frameworks. These are summarised in Table 2, which also presents information about the internal consistency values of the different scales, all of which were acceptable (≥ 0.7). The correlation between these measures, in addition to mean values and SD for subgroups of interest, are presented in Table 3. Measure selection in the #BeeWell study was driven by an extensive consultation process in which more than 150 adolescents were engaged in a series of workshops designed to elicit their understanding of wellbeing. These workshops were combined with inputs from a Questionnaire Advisory Group of academics, mental health professionals, healthcare representatives, education experts, parents, teachers, and adolescents, to inform survey content and measure selection [49].

**Table 2:** Wellbeing Indicators and Measures

Domain	Theoretical Framework	Measure	N of items	Sample Item	Response Format	Cronbach’s alpha
Life satisfaction	Hedonic	Office for National Statistics Life Satisfaction item [50]	1	Overall, how satisfied are you with your life nowadays?	0–10 scale, with 0 = not at all, 10 = completely	
Positive affect	Hedonic	Positive and Negative Affect Scale (positive affect subscale) [51]	5	Indicate to what extent you have felt happy during the past few weeks	Very slightly or not at all, a little, moderately, quite a bit, extremely	0.92
Negative affect	Hedonic	Me and My Feelings (negative affect items from emotional difficulties subscale) [52]	5	I am unhappy	Never, sometimes, always	0.85
Symptoms of distress	Complete state	Me and My Feelings (emotional difficulties subscale) [52]	10	I cry a lot	Never, sometimes, always	0.88
Mental wellbeing	Complete state	Short Warwick Edinburgh Mental Wellbeing Scale [53]	7	I’ve been thinking clearly	None of the time, rarely, some of the time, often, all of the time	0.86
Autonomy	Eudaimonic	Basic Psychological Need Satisfaction and Frustration Scales [54]	6	I generally feel free to express my ideas and opinions	1–5 scale, with 1 = completely not true, 5 = completely true	0.70
Self-Esteem	Eudaimonic	Rosenberg Self-Esteem Scale (positive self-esteem subscale, child version) [55]	5	I am a person of value	Strongly agree, agree, disagree, strongly disagree	0.90
Optimism	Eudaimonic	EPOCH measure (optimism subscale) [56]	4	I think good things are going to happen to me	Almost never, sometimes, often, very often, always	0.81
Positive relations with others	Eudaimonic	Child and Youth Resilience Measure (friendships and social support subscale) [57]	4	I get along with people around me	Not at all, a little, somewhat, quite a bit, a lot	0.84

^a^ 5 specific negative affect items (unhappy, lonely, worry, worry at school, scared) extracted and validated via confirmatory factor analysis (x^2^: 2704.908; d.f.: 5; RMSEA: 0.125; CFI: 0.982; TLI: 0.965)

**Table 3:** Descriptive Analysis: Correlation between Wellbeing Domains, and Mean and Standard Deviation for Subgroups of Interest. Part A. Correlation between Wellbeing Domains

	Life Satisfaction	Positive Affect	Negative Affect	Autonomy	Optimism	Self-Esteem	Positive relationships	Mental Wellbeing	Symptoms of distress
Life Satisfaction	1.00								
Positive Affect	0.64	1.00							
Negative Affect	-0.61	-0.54	1.00						
Autonomy	0.62	0.51	-0.54	1.00					
Optimism	0.59	0.59	-0.49	0.52	1.00				
Self-Esteem	0.62	0.58	-0.58	0.53	0.58	1.00			
Positive relationships	0.48	0.50	-0.42	0.45	0.44	0.45	1.00		
Mental Wellbeing	0.67	0.65	-0.60	0.59	0.66	0.65	0.53	1.00	
Symptoms of distress	-0.62	-0.55	0.94	-0.56	-0.51	-0.60	-0.45	-0.61	1.00

**Table 3:** Part B. Mean and Standard Deviation for Subgroups of Interest (I)

	Life Satisfaction			Positive Affect			Negative Affect			Autonomy			Optimism		
	(0 to 10)			(4 to 10)			(0 to 10)			(6 to 30)			(0 to 20)		
	n	Mean	S.D.	n	Mean	S.D.	n	Mean	S.D.	n	Mean	S.D.	n	Mean	S.D.
Year 8	18,266	6.90	2.47	18,099	13.61	4.06	18,097	3.36	2.52	17,616	20.14	4.58	17,411	11.97	3.76
Year 10	16,052	6.32	2.49	15,979	12.74	4.02	15,930	3.56	2.65	15,721	19.76	4.50	15,594	11.52	3.83
No FSM	25,368	6.70	2.45	25,128	13.29	4.00	25,110	3.46	2.55	24,667	20.01	4.52	24,470	11.82	3.77
FSM	8,263	6.34	2.66	8,258	12.89	4.27	8,232	3.45	2.67	7,996	19.76	4.63	7,868	11.53	3.88
No SEN	29,286	6.62	2.48	29,024	13.21	4.02	29,029	3.45	2.58	28,454	19.98	4.55	28,235	11.77	3.78
SEN	4,461	6.56	2.68	4,484	13.03	4.38	4,434	3.52	2.64	4,323	19.76	4.52	4,211	11.56	3.93
White ethnicity	22,394	6.58	2.50	22,277	13.07	4.03	22,205	3.63	2.59	21,865	19.95	4.47	21,607	11.48	3.75
Black ethnicity	1,666	6.64	2.51	1,648	13.72	4.19	1,653	3.16	2.55	1,600	19.55	4.65	1,576	12.82	3.85
Asian ethnicity	6,041	6.79	2.51	5,931	13.47	4.15	5,964	2.98	2.52	5,803	20.28	4.73	5,771	12.33	3.80
Chinese ethnicity	279	6.20	2.22	279	12.73	3.80	280	4.02	2.51	269	18.70	4.16	270	10.90	3.44
Other ethnicity	744	6.60	2.59	741	13.43	4.20	743	3.19	2.58	713	19.88	4.71	720	12.55	3.91
Mixed ethnicity	1,911	6.46	2.56	1,900	13.21	4.14	1,892	3.39	2.57	1,843	19.70	4.61	1,826	11.82	3.87
Unclassified ethnicity	597	6.48	2.59	610	13.09	4.24	610	3.29	2.53	566	19.68	4.79	563	11.83	4.07
Boy (incl.trans)	14,842	7.23	2.27	14,782	13.98	3.85	14,721	2.57	2.29	14,476	20.69	4.30	14,367	12.50	3.71
Girl (incl.trans)	14,397	6.22	2.48	14,272	12.67	4.00	14,257	4.18	2.47	13,978	19.57	4.56	13,888	11.31	3.69
Non-binary gender identity	836	4.77	2.94	839	10.85	4.50	843	5.56	2.93	813	17.00	4.76	814	9.41	3.87
Other gender identity	988	5.37	2.87	966	11.52	4.37	974	4.89	3.00	954	17.82	4.98	941	10.10	4.03
Prefer not to say gender identity	1,820	6.30	2.70	1,795	12.83	4.43	1,783	3.62	2.72	1,743	19.24	4.61	1,719	11.19	3.92
Heterosexual	24,165	6.94	2.33	23,953	13.61	3.92	23,938	3.06	2.38	23,521	20.49	4.37	23,342	12.22	3.68
Gay/Lesbian	969	4.80	2.73	959	10.80	4.15	950	5.65	2.69	942	17.46	4.61	931	9.20	3.72
Bisexual/Pansexual	2,773	4.90	2.54	2,768	10.92	3.87	2,757	5.72	2.41	2,707	17.18	4.38	2,700	9.45	3.47
Prefer not to say sexual identity	3,162	6.38	2.60	3,163	12.91	4.20	3,138	3.85	2.66	3,051	19.29	4.53	3,024	11.28	3.83
Other sexual identity	1,316	6.01	2.74	1,308	12.46	4.35	1,300	4.15	2.86	1,272	18.75	4.85	1,265	10.96	4.00
Cisgender	28,520	6.76	2.40	28,334	13.37	3.96	28,259	3.33	2.48	27,751	20.19	4.44	27,554	11.95	3.73
Transgender	2,525	5.21	2.92	2,502	11.40	4.46	2,517	5.08	3.04	2,452	17.59	4.94	2,438	9.98	4.03

n = observations; S.D. = Standard Deviation; FSM = eligible for free-school meals; SEN = special educational needs

**Table 3:** Mean and Standard Deviation for Subgroups of Interest (II)

	Self-Esteem			Positive relationships			Mental Wellbeing			Symptoms of distress		
	(5 to 20)			(4 to 20)			(7 to 35)			(0 to 20)		
	n	Mean	S.D.	n	Mean	S.D.	n	Mean	S.D.	n	Mean	S.D.
Year 8	17,577	14.75	3.42	18,046	15.52	3.60	17,198	23.47	5.81	17,728	6.60	4.63
Year 10	15,643	14.19	3.45	15,920	15.14	3.65	15,435	22.65	5.79	15,621	6.83	4.83
No FSM	24,570	14.54	3.41	25,083	15.47	3.55	24,188	23.22	5.73	24,619	6.67	4.67
FSM	7,976	14.22	3.55	8,202	14.95	3.84	7,788	22.51	6.06	8,054	6.91	4.91
No SEN	28,359	14.50	3.43	28,898	15.43	3.58	27,980	23.14	5.79	28,479	6.69	4.72
SEN	4,301	14.25	3.55	4,503	14.75	3.89	4,098	22.43	5.98	4,314	6.95	4.79
White	21,727	14.26	3.43	22,217	15.20	3.63	21,403	22.79	5.77	21,794	7.04	4.75
Black ethnicity	1,588	15.23	3.44	1,604	15.65	3.58	1,573	23.83	5.78	1,601	6.12	4.56
Asian ethnicity	5,832	14.93	3.42	5,969	15.81	3.60	5,656	23.72	5.97	5,826	5.88	4.62
Chinese ethnicity	271	13.86	3.14	271	14.93	3.51	271	22.51	5.27	271	7.46	4.47
Other ethnicity	718	14.95	3.46	743	15.41	3.65	691	24.02	5.83	732	6.30	4.75
Mixed ethnicity	1,819	14.47	3.59	1,888	15.33	3.65	1,802	22.95	5.88	1,850	6.65	4.71
Unclassified	582	14.55	3.65	593	15.07	3.70	563	22.96	6.07	602	6.47	4.71
Boy (incl.trans)	14,512	15.28	3.24	14,724	15.46	3.53	14,204	24.61	5.43	14,493	5.01	4.09
Girl (incl.trans)	13,881	13.94	3.32	14,227	15.53	3.52	13,717	22.10	5.62	13,934	8.06	4.51
Non-binary gender	816	11.96	4.06	838	13.37	4.16	810	18.84	6.53	824	10.99	5.47
Other gender	941	12.63	4.15	969	13.79	4.24	912	19.94	6.40	956	9.71	5.61
Prefer not to say gender	1,736	13.99	3.72	1,781	14.50	4.04	1,680	21.83	6.22	1,742	7.21	5.01
Heterosexual	23,433	14.94	3.21	23,881	15.71	3.43	23,065	23.89	5.52	23,503	5.93	4.30
Gay/Lesbian sexuality	930	11.81	3.82	946	13.55	4.16	908	18.95	6.08	932	11.05	4.95
Bisexual/Pansexual sexuality	2,699	11.93	3.48	2,737	13.80	3.83	2,670	19.09	5.45	2,694	11.14	4.44
Prefer not to say sexuality	3,053	14.15	3.52	3,167	14.75	3.87	2,956	22.08	5.96	3,058	7.51	4.83
Other sexuality	1,288	13.73	3.84	1,311	14.34	3.95	1,262	21.48	6.10	1,273	8.23	5.27
Cisgender	27,686	14.67	3.31	28,229	15.53	3.50	27,231	23.44	5.62	27,729	6.43	4.51
Transgender	2,441	12.51	4.17	2,506	13.74	4.20	2,395	19.74	6.56	2,459	10.04	5.68

n = observations; S.D. = Standard Deviation; FSM = eligible for free-school meals; SEN = special educational needs

When considering negative affect (hedonic wellbeing framework), we used a subset of ‘pure’ negative affect items (e.g., those pertaining specifically to the frequency with which negative emotions, such as feeling worried, are experienced) from the Me and My Feelings scale [52], whereas when considering symptoms of distress (complete state wellbeing framework), we used the full range of items (including those that pertain to the frequency with which somatic symptoms, such as having problems sleeping, are experienced). With the exception of this particular measure, we sought to avoid measure overlap across theoretical frameworks in order to avoid uninterpretable findings regarding the scope and scale of wellbeing inequalities.

#### Covariates

A range of variables representing different participant socio-demographic characteristics were included as co-variates in our models, as follows: age/year group; free school meal eligibility in the last six years (FSM); SEN; and, ethnicity. These data were drawn from an administrative dataset provided by the 10 Greater Manchester Local Authorities, and were included in order to increase the robustness and precision of our estimates relating to the focal variables of interest (e.g., those pertaining to gender and sexual identity) by accounting for the influence of other characteristics for which there are established wellbeing inequalities [58].

### Procedure

#### Ethical approval

from the authors’ host institution’s University Research Ethics Committee was sought and granted prior to the commencement of data collection (Ref: 2021-11133-18179). Consistent with the conditions of this approval, opt-out parent/carer consent and young people’s assent to participate was used in combination for informed consent, leading to n = 363 not completing surveys (0.95% of a total of 363 + 37,978 = 38,341). Surveys were administered en masse to adolescents in school settings, supported by school staff (who provided standardised instructions), via a secure online survey platform (Qualtrics). Measures were presented in a random order to spread missing data due to item fatigue evenly across the survey.

#### Analytic strategy

**Fig. 1:**
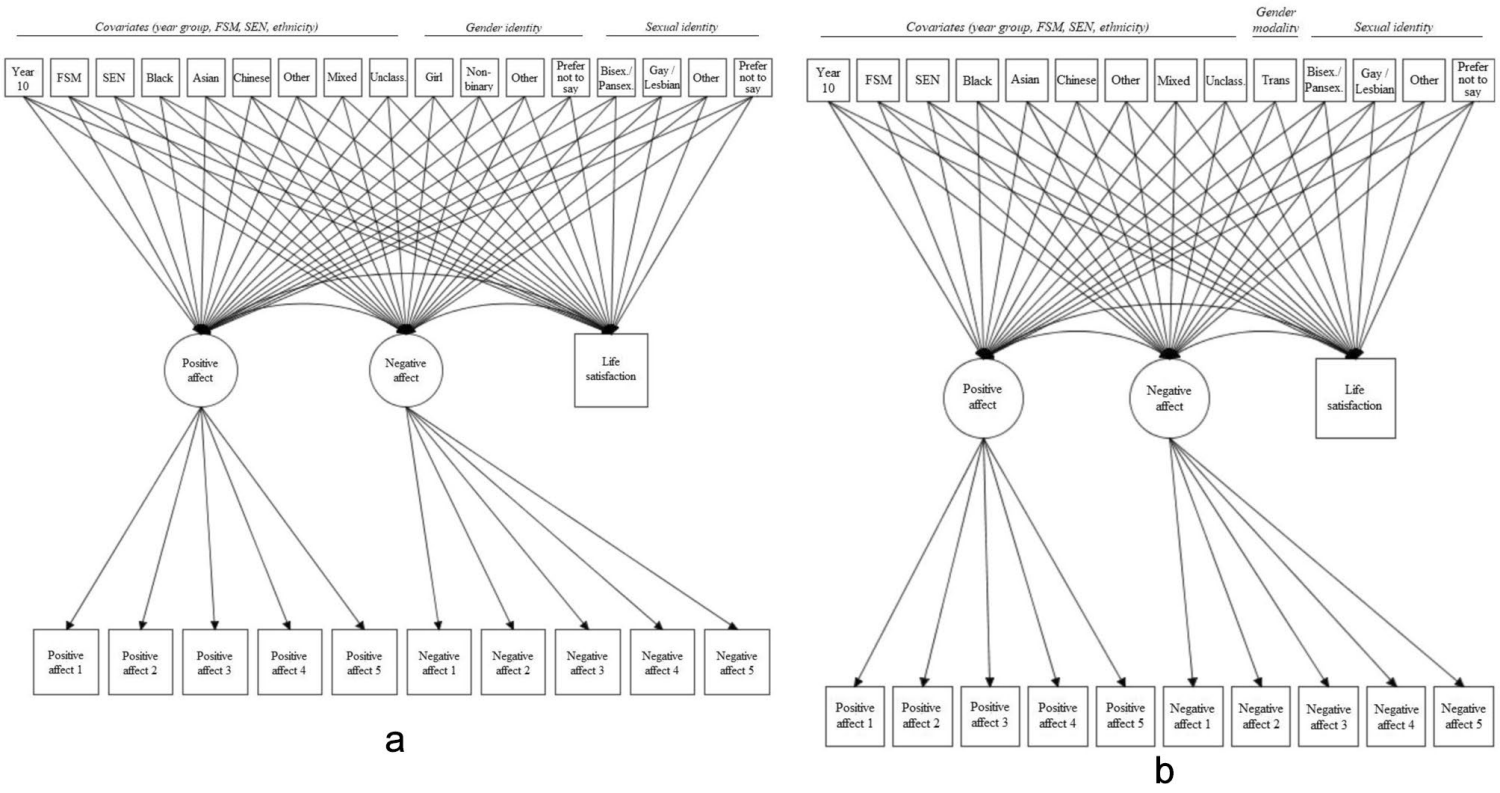
(**A**) Hedonic Wellbeing Model A. (**B**) Hedonic Wellbeing Model B

Before estimating structural models, as a preliminary analysis, we first compared three possible measurement structures (unidimensional, bifactor, correlated factors) for each wellbeing framework. This preliminary analysis is explained in detail in Appendix 1 (Part 1). In brief, models were compared on a balance of fit, interpretability and dimensionality indices [59, 60]. Based on this, we determined that the most appropriate measurement structure for each model was *correlated factors* (in which theorised wellbeing domains are modelled as distinct but correlated factors). We subsequently adopted the following strategy to estimate structural correlated factors models to assess gender and sexual identity wellbeing inequalities. For each framework (hedonic, eudaimonic, and complete state), we estimated two models. Model A included all covariates (year group, FSM, SEN, ethnicity), gender identity, and sexual identity. Multi-categorical variables (ethnicity, gender identity, and sexual identity) were dummy coded (e.g., white: no = 0, yes = 1; black: no = 0, yes = 1; *et cetera*) with the reference category (white for ethnicity; boy for gender identity; heterosexual for sexual identity) omitted. Comparisons were therefore made between each dummy minority group (e.g., gay/lesbian) and the reference category (e.g., heterosexual). In Model B we estimated the same model, replacing all the gender identity variables with the gender modality (transgender/cisgender) variable. The motivation for fitting gender identity and gender modality in separate models (and, indeed, for not including sex as a covariate in Model A) was to avoid multicollinearity effects because, in our sample, the majority (nearly 80%) of adolescents’ self-reported gender identity was the same as their sex. Figure 1 A and 1B illustrate this for the hedonic Models A and B, respectively.

Analyses were conducted in MPlus 8.7. The clustered/hierarchical nature of the dataset (adolescents nested within schools) was taken into account using the ‘Type = complex’ function which adjusts standard errors to account for dependence in the data. It was considered appropriate to treat measures as continuous given that all except Me and My Feelings (negative affect and symptoms of distress) had five response categories or more and did not exhibit substantial asymmetry [61]. We therefore used the maximum likelihood with robust standard errors (MLR) estimator. Where appropriate, items from different scales were reversed to ensure all of them had the same orientation. Missing data were accounted for using full information maximum likelihood (FIML). Given the response format of the Me and My Feelings measure, we conducted sensitivity analyses of the hedonic and complete state models using the weighted least square mean and variance adjusted (WLSMV) estimator, with multiple imputation (20 imputed datasets) to account for missing data (as FIML is not available in WLSMV). These analyses (see Tables A1.2 and A1.3 in Appendix 1, Part 2) produced very similar estimates to those produced using the MLR estimator, with negligible differences in path coefficients. Model fit was judged in line with established cut-offs, with values > = 0.95 for the Comparative Fit Index (CFI), <= 0.06 for the root mean square error of approximation (RMSEA), and < = 0.08 for the standardized root mean square residual (SRMR) considered indicative of good fit [59].

**Fig. 2:**
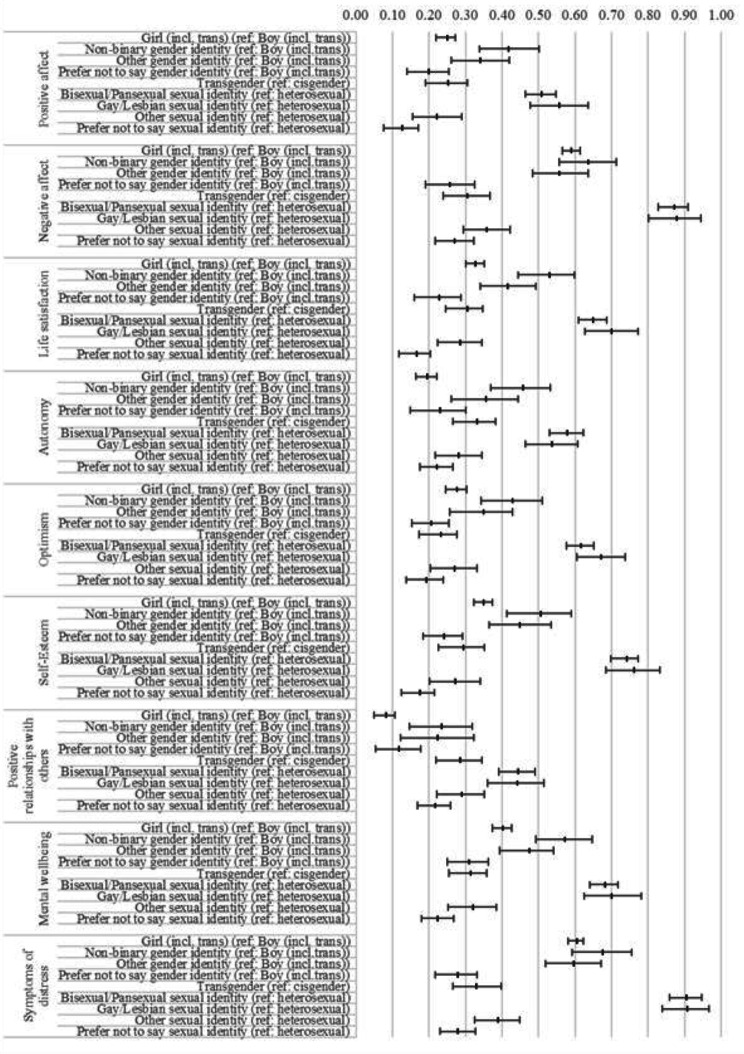
Visualisation of structural path co-efficients (and their confidence intervals) within and between wellbeing subdomains

Finally, given the very large sample size, most or all structural paths were expected to be statistically significant [60]. We therefore opted to focus on the relative magnitude of the path coefficients within and across models. Our interpretation was guided by overlap/non-overlap in confidence intervals (CIs) between the different structural path coefficients (see Fig. 2). More broadly, in the absence of any agreed thresholds for what size of inequality might be considered to be practically significant or substantively important, we drew on two very large, wide-ranging analyses of predictors of self-reported adolescent wellbeing [12, 62], using these to contextualize our findings (see Discussion).

## Results

### Descriptive information of the study sample and variables of interest

The demographic characteristics of the study sample are summarised in Table 1, alongside national averages, where available. From this, one can surmise that the study sample mirrors the national picture for secondary-aged students with respect to sex, year group/age, and special educational needs. Ethnicity profiles are also similar, with the exceptions of the study sample containing a relatively larger proportion of Asian adolescents and relatively smaller proportion of White adolescents than the national average (mirroring the school-level pattern noted in the preceding subsection). However, the study sample mirrors the population of adolescents aged 11–16 in Greater Manchester very closely, with far less deviation in terms of ethnic composition than that noted above [58].

Table 2 shows information about the internal consistency of the wellbeing scales used in this study. Inspection of Table 3 (descriptive statistics) reveals that expected correlations between wellbeing domains were borne out in the data, both in terms of direction (all positively associated, with the exception of negative affect and symptoms of distress) and magnitude (mostly moderate, or moderate-to-strong, indicating related but distinct constructs). Mean values and SDs for the subgroups of interest are indicative of a pattern in which gender and sexual identity categories produce the most substantial disparities, relative to other characteristics such as ethnicity.

### Key findings

Table 4 provides fit indices for the structural correlated factors models, which indicate good model fit. Tables 5 and 6, and 7 present the results of models corresponding to the hedonic, eudaimonic, and complete state frameworks, respectively. Figure 2 provides a visualisation of path coefficients and their CIs, with an accompanying online version available that enables readers to filter by outcome and group for easier comparison (see Supplementary Materials). In both the tables and figure, the size of structural paths, representing wellbeing inequalities, are reported in standard deviations (SD; e.g., bisexual/pansexual adolescents had estimated life satisfaction scores 0.65 SD lower than their heterosexual peers). In Fig. 2, the size of these structural paths are also reported in *absolute* terms to facilitate comparisons between measures where paths have opposite signs (e.g., negative affect and optimism). Below we note substantive patterns derived from the above, presented as a series of Key Findings that match our research questions in the interest of clarity.

**Table 4:** Model fit for the structural models

			Model fit indices				
Model	Scales	Type	x^2^	d.f.	RMSEA	CFI	SRMR
Hedonic	Life satisfaction, positive affect, negative affect	A	9230	178	0.038	0.958	0.024
		B	7763	154	0.040	0.960	0.024
Eudaimonic	Autonomy, optimism, self-esteem, positive relationships	A	19,902	401	0.037	0.924	0.036
		B	16,927	356	0.038	0.928	0.036
Complete state	Mental wellbeing, symptoms of distress	A	20,459	373	0.039	0.913	0.028
		B	15,919	328	0.039	0.924	0.027

**Table 5:** SEM estimates for gender identity, sexual identity, and covariates (hedonic model)

	Positive Affect						Negative Affect						Life satisfaction					
	Model A			Model B			Model A			Model B			Model A			Model B		
	B		S.E.	B		S.E.	B		S.E.	B		S.E.	B		S.E.	B		S.E.
Year 10 (ref: year 8)	-0.22	***	0.01	-0.22	***	0.02	0.10	***	0.02	0.11	***	0.02	-0.24	***	0.01	-0.25	***	0.01
FSM (ref: no FSM)	-0.12	***	0.01	-0.12	***	0.01	0.00		0.01	0.00		0.02	-0.14	***	0.01	-0.14	***	0.01
SEN (ref: no SEN)	-0.07	**	0.02	-0.03		0.02	0.05	*	0.02	-0.03		0.02	-0.01		0.02	0.04	*	0.02
Black ethnicity (ref: white)	0.11	***	0.03	0.13	***	0.03	-0.13	***	0.03	-0.12	***	0.03	-0.01		0.03	-0.01		0.03
Asian ethnicity (ref: white)	0.05	*	0.02	0.03		0.02	-0.21	***	0.02	-0.19	***	0.03	0.04	*	0.02	0.02		0.02
Chinese ethnicity (ref: white)	-0.02		0.06	-0.01		0.05	0.07		0.06	0.08		0.06	-0.16	*	0.07	-0.19	**	0.06
Other ethnicity (ref: white)	0.05		0.04	0.06		0.04	-0.11	**	0.04	-0.17	**	0.05	0.01		0.04	0.03		0.04
Mixed ethnicity (ref: white)	0.03		0.03	0.01		0.03	-0.07	*	0.03	-0.04		0.03	-0.06	*	0.03	-0.07	*	0.03
Unclassified ethnicity (ref: white)	-0.01		0.05	-0.03		0.05	-0.13	**	0.04	-0.11	*	0.05	-0.04		0.05	-0.08		0.05
Girl (incl. trans) (ref: Boy (incl. trans))	-0.25	***	0.01				0.59	***	0.01				-0.33	***	0.01			
Non-binary gender identity (ref: Boy (incl.trans))	-0.42	***	0.04				0.64	***	0.04				-0.53	***	0.04			
Other gender identity (ref: Boy (incl.trans))	-0.34	***	0.04				0.55	***	0.04				-0.41	***	0.04			
Prefer not to say gender identity (ref: male (incl.trans))	-0.20	***	0.03				0.25	***	0.03				-0.23	***	0.03			
Bisexual/Pansexual (ref: heterosexual)	-0.51	***	0.02	-0.56	***	0.02	0.87	***	0.02	0.99	***	0.02	-0.65	***	0.02	-0.72	***	0.02
Gay/Lesbian (ref: heterosexual)	-0.56	***	0.04	-0.56	***	0.05	0.88	***	0.04	0.91	***	0.04	-0.70	***	0.04	-0.73	***	0.04
Prefer not to say sexual identity (ref: heterosexual)	-0.12	***	0.02	-0.19	***	0.02	0.27	***	0.03	0.39	***	0.03	-0.16	***	0.02	-0.23	***	0.02
Other sexual identity (ref: heterosexual)	-0.22	***	0.03	-0.23	***	0.04	0.36	***	0.03	0.36	***	0.04	-0.28	***	0.03	-0.29	***	0.04
Transgender (ref: cisgender)				-0.25	***	0.03				0.30	***	0.03				-0.30	***	0.03

**Table 6:** SEM estimates for gender identity, sexual identity and covariates (eudaimonic model)

	Autonomy						Optimism						Self-Esteem						Positive relationships					
	Model A			Model B			Model A			Model B			Model A			Model B			Model A			Model B		
	B		S.E.	B		S.E.	B		S.E.	B		S.E.	B		S.E.	B		S.E.	B		S.E.	B		S.E.
Year 10 (ref: year 8)	-0.10	***	0.01	-0.11	***	0.01	-0.13	***	0.01	-0.13	***	0.01	-0.18	***	0.01	-0.19	***	0.01	-0.14	***	0.01	-0.15	***	0.01
FSM (ref: no FSM)	-0.09	***	0.01	-0.10	***	0.01	-0.10	***	0.01	-0.10	***	0.02	-0.10	***	0.01	-0.10	***	0.01	-0.13	***	0.01	-0.12	***	0.01
SEN (ref: no SEN)	-0.06	**	0.02	-0.03		0.02	0.00		0.02	0.04		0.02	-0.04		0.02	0.01		0.02	-0.12	***	0.02	-0.14	***	0.02
Black ethnicity (ref: white)	-0.11	***	0.03	-0.11	***	0.03	0.38	***	0.03	0.38	***	0.03	0.25	***	0.03	0.24	***	0.03	0.11	**	0.03	0.10	**	0.03
Asian ethnicity (ref: white)	-0.03		0.02	-0.04	*	0.02	0.20	***	0.02	0.18	***	0.03	0.15	***	0.02	0.13	***	0.02	0.15	***	0.02	0.15	***	0.02
Chinese ethnicity (ref: white)	-0.21	**	0.06	-0.23	**	0.07	-0.15	*	0.07	-0.14	*	0.07	-0.11		0.06	-0.11	*	0.05	-0.09		0.07	-0.06		0.07
Other ethnicity (ref: white)	-0.06		0.05	-0.04		0.05	0.25	***	0.05	0.30	***	0.06	0.16	***	0.04	0.19	***	0.04	0.06		0.04	0.06		0.05
Mixed ethnicity (ref: white)	-0.08	**	0.03	-0.09	**	0.03	0.09	**	0.03	0.09	**	0.03	0.04		0.03	0.03		0.03	0.03		0.03	0.03		0.03
Unclassified ethnicity (ref: white)	-0.08		0.05	-0.11	*	0.06	0.08		0.05	0.06		0.05	0.08		0.05	0.06		0.05	-0.05		0.05	-0.05		0.05
Girl (incl. trans) (ref: Boy (incl. trans))	-0.19	***	0.01				-0.27	***	0.02				-0.35	***	0.01				0.08	***	0.02			
Non-binary gender identity (ref: Boy (incl.trans))	-0.46	***	0.04				-0.43	***	0.04				-0.50	***	0.05				-0.23	***	0.05			
Other gender identity (ref: Boy (incl.trans))	-0.35	***	0.05				-0.35	***	0.04				-0.45	***	0.04				-0.22	***	0.05			
Prefer not to say gender identity (ref: male (incl.trans))	-0.23	***	0.04				-0.20	***	0.03				-0.24	***	0.03				-0.12	***	0.03			
Bisexual/Pansexual (ref: heterosexual)	-0.58	***	0.02	-0.62	***	0.02	-0.61	***	0.02	-0.68	***	0.02	-0.74	***	0.02	-0.82	***	0.02	-0.44	***	0.03	-0.41	***	0.03
Gay/Lesbian (ref: heterosexual)	-0.53	***	0.04	-0.53	***	0.04	-0.67	***	0.03	-0.71	***	0.04	-0.76	***	0.04	-0.77	***	0.04	-0.44	***	0.04	-0.41	***	0.04
Prefer not to say sexual identity (ref: heterosexual)	-0.22	***	0.02	-0.28	***	0.02	-0.19	***	0.03	-0.27	***	0.03	-0.17	***	0.02	-0.24	***	0.02	-0.22	***	0.02	-0.22	***	0.03
Other sexual identity (ref: heterosexual)	-0.28	***	0.03	-0.26	***	0.03	-0.27	***	0.03	-0.27	***	0.04	-0.27	***	0.04	-0.28	***	0.04	-0.29	***	0.04	-0.27	***	0.04
Transgender (ref: cisgender)				-0.33	***	0.03				-0.23	***	0.03				-0.29	***	0.03				-0.28	***	0.03

Standardized coefficients; *** p ≤ 0.001, ** p ≤ 0.01, * p ≤ 0.05

**Table 7:** SEM estimates for gender identity, sexual identity and covariates (complete state model)

	Mental wellbeing						Symptoms of distress					
	Model A			Model B			Model A			Model B		
	B		S.E.	B		S.E.	B		S.E.	B		S.E.
Year 10 (ref: year 8)	-0.16	***	0.01	-0.18	***	0.01	0.07	***	0.01	0.08	***	0.02
FSM (ref: no FSM)	-0.12	***	0.01	-0.12	***	0.01	0.04	**	0.01	0.05	**	0.01
SEN (ref: no SEN)	-0.09	***	0.02	-0.02		0.02	0.07	***	0.02	-0.02		0.02
Black ethnicity (ref: white)	0.15	***	0.03	0.13	***	0.03	-0.13	***	0.03	-0.13	***	0.03
Asian ethnicity (ref: white)	0.14	***	0.02	0.13	***	0.03	-0.19	***	0.02	-0.17	***	0.03
Chinese ethnicity (ref: white)	-0.01		0.06	-0.01		0.05	0.04		0.06	0.05		0.06
Other ethnicity (ref: white)	0.18	***	0.04	0.20	***	0.04	-0.11	**	0.04	-0.16	**	0.05
Mixed ethnicity (ref: white)	0.01		0.03	0.01		0.03	-0.06	*	0.03	-0.04		0.03
Unclassified ethnicity (ref: white)	0.04		0.06	0.02		0.07	-0.12	**	0.04	-0.10	*	0.05
Girl (incl. trans) (ref: Boy (incl. trans))	-0.40	***	0.01				0.60	***	0.01			
Non-binary gender identity (ref: Boy (incl.trans))	-0.57	***	0.04				0.67	***	0.04			
Other gender identity (ref: Boy (incl.trans))	-0.47	***	0.04				0.60	***	0.04			
Prefer not to say gender identity (ref: male (incl.trans))	-0.31	***	0.03				0.28	***	0.03			
Bisexual/Pansexual (ref: heterosexual)	-0.68	***	0.02	-0.78	***	0.02	0.90	***	0.02	1.03	***	0.02
Gay/Lesbian (ref: heterosexual)	-0.70	***	0.04	-0.74	***	0.04	0.91	***	0.04	0.94	***	0.04
Prefer not to say sexual identity (ref: heterosexual)	-0.22	***	0.02	-0.31	***	0.02	0.28	***	0.02	0.40	***	0.03
Other sexual identity (ref: heterosexual)	-0.32	***	0.03	-0.34	***	0.04	0.39	***	0.03	0.40	***	0.04
Transgender (ref: cisgender)				-0.31	***	0.03				0.33	***	0.03

Standardized coefficients; *** p ≤ 0.001, ** p ≤ 0.01, * p ≤ 0.05

#### Key finding 1: adolescent gender and sexual identity wellbeing inequalities are routinely more substantial than those pertaining to other characteristics (RQ1)

Across all frameworks and domains within them, the magnitude of wellbeing inequalities pertaining to gender and sexual identity are routinely greater than those pertaining to covariates (age, FSM, SEN, and ethnicity). Thus, in all but a few cases, the *smallest* gender identity or sexual identity inequality is *greater* than the largest covariate inequality. Furthermore, almost all cases where a covariate inequality was equivalent to or exceeded the magnitude of a gender identity/sexual identity inequality involved the ‘prefer not to say’ categories in the latter (e.g., in hedonic Model A, adolescents who prefer not to report their sexual identity had estimated positive affect scores of 0.12 SD lower than heterosexual adolescents; in the same analysis, those in Year 10 had estimated positive affect scores 0.22 SD lower than those in Year 8).

#### Key finding 2: gender identity wellbeing inequalities follow a consistent pattern, with the largest disparities evident between gender diverse adolescents and boys (RQ2)

Model A in each wellbeing framework reveals that point estimates are consistent in the order of magnitude for gender (though there is some overlap of CIs across predictors), as follows (with the illustrative example of mental wellbeing in the complete state model): non-binary (largest, β= − 0.57); describe themselves in another way (β=− 0.47); girls (β=− 0.40); and, prefer not to say (smallest, β= − 0.31). Model B in each wellbeing framework consistently reveals inequalities for transgender adolescents when compared to their cisgender counterparts (β=− 0.31 in the case of mental wellbeing). Reading back across to Model A, the observed wellbeing inequalities for transgender adolescents consistently places them at or around the smallest order of magnitude (e.g., ≈ β= 0.3), though of course the difference in reference group should be noted (e.g., boys in Model A, cisgender in Model B).

#### Key finding 3: sexual identity wellbeing inequalities follow a consistent pattern, with the largest disparities evident between sexual minority adolescents and their heterosexual peers (RQ3)

Model A in each wellbeing framework reveals that point estimates are consistent in the order of magnitude for gender (though there is some overlap of CIs), as follows (with the illustrative example of self-esteem in the eudaimonic model): gay/lesbian (largest, β= − 0.76); bi/pansexual (β=− 0.74); describe in another way (β=− 0.27); and, prefer not to say (smallest, β= − 0.17). Of note is the fact that the magnitude of wellbeing inequalities for bi/pansexual and gay/lesbian adolescents are remarkably similar, with overlap between CIs across all domains.

#### Key finding 4: adolescent sexual identity wellbeing inequalities across subgroups of interest are consistent, irrespective of how gender identity is categorised (RQ4)

Contrasting Model A and B in each wellbeing domain, we can see that substituting our gender identity variable (A) with our gender modality variable (B) does not substantively impact the magnitude of the structural paths for sexual identity, with trivial coefficient changes in all cases except for those who prefer not to report their sexual identity. Using symptoms of distress in the complete state models as an example, being gay/lesbian (compared to heterosexual) is associated with an estimated 0.91 SD increase in scores in Model A, and estimated 0.94 SD increase in scores in Model B.

#### Key finding 5: adolescent gender and sexual identity wellbeing inequalities vary across frameworks and domains (RQ5)

Reading across and within our three substantive wellbeing frameworks (hedonic, eudaimonic, complete state), there are three related patterns. First, the most substantial inequalities pertain to negative affect (hedonic) and symptoms of distress (complete state), both of which are derived from the Me and My Feelings emotional difficulties subscale [52], with some shared items (see Measures section). For girls, bi/pansexual, and gay/lesbian respondents, there are no CI overlaps between path coefficients for these versus other wellbeing domains, with a single exception (self-esteem for gay/lesbian respondents). Second, the eudaimonic framework hosts the smallest wellbeing inequality: positive relationships with others. For girls, bi/pansexual, gay/lesbian, and non-binary respondents, there are only two CI overlaps between path coefficients for this versus other wellbeing domains (positive affect for bi/pansexual and gay/lesbian respondents; autonomy for gay/lesbian respondents). Third, variability is evident within the three wellbeing frameworks, with (for example) substantially greater disparities in symptoms of distress than mental wellbeing for girls, bisexual, and gay/lesbian adolescents in the complete state models.

## Discussion

In the current study we sought to examine wellbeing inequalities relating to gender and sexual identity among adolescents via analysis of the #BeeWell dataset. Doing so enabled a novel contribution to knowledge through a wide-ranging investigation of multiple wellbeing frameworks and domains that went beyond the traditional focus on symptoms of distress; included segmented analyses by sexual *and* gender identity; controlled for a range of socio-demographic covariates for which there are established wellbeing inequalities (something frequently not attended to in comparable studies [63, 64]); and, updated and extended the evidence base to England, in relation to one of the most neglected stages of development: early adolescence.

After determing the most appropriate measurement structure for each wellbeing framework (correlated factors), our structural models revealed that the magnitude of wellbeing inequalities pertaining to gender and sexual identity were routinely greater than those concerning ethnicity, socio-economic disadvantage, age, and special educational needs. For gender identity, the largest wellbeing inequalities were evident for those adolescents who identified as non-binary, followed by those who described themselves in another way, girls, and finally, those who preferred not to say (all compared to boys). In a parallel analysis, wellbeing inequalities were also found for transgender youth (compared to their cisgender peers). Regarding sexual identity, the greatest wellbeing inequalities were apparent for those who identified as gay/lesbian or bi/pansexual, followed by those who described themselves in another way, and finally, those who preferred not to say (all compared to those identifying as heterosexual).

Reading across and within our three wellbeing frameworks (hedonic, eudaimonic, complete state), there were three related patterns. First, the most substantial inequalities pertained to negative affect (hedonic) and symptoms of distress (complete state), both of which were operationalized using items from the same measure (Me and My Feelings). Second, the smallest wellbeing inequality was for positive relationships with others. Finally, variability was evident within the three wellbeing frameworks, with, for example, substantially greater disparities in symptoms of distress than mental wellbeing in the complete state models.

Adolescents whose identities transcend traditional boundaries (i.e., those who are non-binary or describe themselves in another way) were subject to the most substantial inequalities when considering gender identity. Given that so little is known about the wellbeing of gender diverse early adolescents, this is an important finding. Identity formation is a crucial developmental task of adolescence, with identity expected to consolidate over time [65]. Early adolescence is therefore a period in which we would expect to see greater exploration of identity options. However, this appears to come at a cost to wellbeing among those whose emergent identity does not conform to social expectations and the conventional boy/girl binary [66].

The same can also be said for the inequalities that were identified in relation to gender modality (i.e. among transgender youth). Here, our findings align with what earlier research has found regarding mental health difficulties [39], and provide new evidence in relation to other facets of wellbeing. We note disparities between cisgender and transgender youth pertaining to all wellbeing domains, which were equivalent in size to that observed for symptoms of distress. Thus, attempts to address this issue should factor in the need to increase feelings of agency, psychological functioning and other aspects of wellbeing (e.g., both feeling good and thriving) alongside the traditional focus on alleviating distress.

While the current study focuses on outcomes rather than mechanisms, our findings are consistent with what would be predicted by minority stress theory [33], and indeed early adolescence may be a particularly sensitive period for exposure to such stressors, given the heightened social comparison and affectivity that characterise this period [67]. Further research is needed to examine the extent to which experiences of prejudice, discrimination, sexual identity concealment, expectations of rejection, and internalised stigma [34–36], and punitive and traumatic reactions from parents and caregivers in response to their children’s identity and/or orientation [37], might explain the magnitude of wellbeing inequalities observed here for gender diverse and sexual minority youth. The existing evidence base is unfortunately rather limited, being focused almost exclusively on distress (neglecting broader aspects of wellbeing), primarily cross-sectional in nature (limiting causal inference), rather narrowly focused on victimisation pertaining to sexual identity (neglecting other minority stressors), and failing to pay attention to how gender and sexual identity intersect in relation to minority stress and wellbeing outcomes (limiting the precision of conclusions drawn) (Authors, under review).

Consistent with prior research [4], we also found evidence of inequalities across the full range of wellbeing outcomes for girls when compared to boys. However, the magnitude of these disparities varied considerably across different wellbeing outcomes. Thus, while the gap between boys and girls was highly pronounced for negative affect and symptoms of distress (and, indeed, was comparable to inequalities for gender diverse youth), it was considerably smaller for autonomy, and negligible for positive relationships. Relatedly, it is noteworthy that, in comparison to outcomes in the hedonic and complete state frameworks, eudaimonic outcomes generally produced the least prominent boy-girl inequalities. This finding is consistent with recent research on eudaimonia in emerging adulthood [68], and underscores the idea that the scale of inequalities can depend very much on how one defines and measures wellbeing [69]. Hence, while we found that girls are disadvantaged compared to boys in terms of eudaimonic wellbeing, they are *more* disadvantaged when their wellbeing is viewed through the lens of complete state or hedonic wellbeing.

In comparing our findings with those of two very large, wide-ranging analyses of predictors of self-reported adolescent wellbeing that span three very large datasets (Monitoring the Future, Youth Risk and Behaviour Survey, and the Millennium Cohort Study) [12, 62], there are several points of note that speak to the practical significance and substantive importance of the inequalities noted above. First, the magnitude of inequalities observed for our co-variates is consistent with previous work (e.g., small and inconsistent ethnicity disparities; small but consistently negative gaps in relation to SEN and socio-economic deprivation [62]). Second, there is also consistency with other work for the magnitude of the boy-girl gap for comparable wellbeing domains (e.g., symptoms of distress [62]). Third, and most importantly, the marked inequalities observed for gender diverse (e.g., non-binary, describe in another way) and sexual minority (e.g., gay/lesbian, bi/pansexual) adolescents in the current study overshadow most of the wide range of factors and characteristics previously shown to be associated with wellbeing by a considerable margin. For example, consider that being bullied (compared to not being bullied) is consistently associated with a ≈ 0.2 SD increase in symptoms of distress in adolescence [12, 62]; the association between being gay/lesbian or bi/pansexual (compared to heterosexual) and symptoms of distress is more than 4x larger (> 0.9 SD, with even the lower CI being > 0.8 SD).

Few would argue that the effects of bullying on wellbeing do not warrant intervention, and indeed its’ prevention and remediation has been rightly flagged as a policy and practice priority [68]. Accordingly, we argue strongly that the scale of the wellbeing disparities is of such substantive importance to justify a comprehensive prevention and intervention response, especially given that said disparities appear to have been amplified over the course of the Covid-19 pandemic (e.g., average of 0.39 SD increase in symptoms of distress among sexual minority youth in pre-pandemic research [63]). It is this issue to which we now turn.

### Implications

Given the findings of the current study, a fundamental shift in measurement approaches is warranted. First, our analyses highlight the need to move beyond a predominant focus on symptoms of distress, to include a range of broader wellbeing indicators. Our analysis show moderate correlations between wellbeing indicators -suggesting that they are not measuring the same thing- and all of them reveal highly important to understand wellbeing vulnerability affecting gender and sexual identity minorities. Second, gender *and* sexual identity should be measured in ways that enable adolescents to report identities that differ from social and cultural norms relating to rigid, binary constructs (e.g., boy vs. girl). This point is underscored by the fact that in the current study, those who reported being gender diverse (e.g., non-binary, describe in another way) were subject to the most marked wellbeing inequalities. Furthermore, it is noteworthy that although there are now national statistics on adult gender diversity and sexual orientation/identity in England [70], there are no equivalent figures for young people (hence, the absence of these figures in Table 1). Within the NHS, guidance exists to improve data collection about gender identity [71], but is often poor quality, leading to experiences of discrimination and unequal treatment [72]. Finally, overall, our findings show the need for effective interventions to support the wellbeing of LGBTQ + adolescents, which has also been highlighted in research investigating interventions for gender and sexual minority groups [73, 74].

### Strengths and limitations

Together with the study strengths highlighted above, there are numerous limitations that need to be borne in mind. First, though self-report is arguably the optimal method for assessing the wellbeing domains that were the focus of our inquiry [75], its exclusive use raises the question as to whether common method variance underpins some of our findings (i.e., that patterns observed across wellbeing domains were partly attributable to the fact that all surveys were self-report and had similar response formats). Second, despite the fact that we were able to comprehensively model the domains of the hedonic and complete state wellbeing frameworks, our reliance on an existing dataset meant that some domains of the eudaimonic framework could not be included (e.g., environmental mastery, personal growth). Third, as the #BeeWell dataset only contained a single wave of data at the point at which the current study was written, analyses focusing on the *development* of wellbeing inequalities, and indeed the factors theorised to underpin them, were not possible. Fourth, the dataset was unbalanced. While this was to be expected, it reduced the precision of our estimates for minority gender and sexual identity groups, as evidenced in wider CIs. Fifth, caution is needed with regards to the generalizability of these results to other populations, as there is considerable variation across societies in institutional (e.g., rules and laws affecting LGBTQ + young people, the type of health care services available) and societal factors (e.g. levels of discrimination, denial of discrimination among gender and sexual orientation/identity minorities) that are relevant to LGBTQ + young people [76, 77].

Relatedly, we also note the aforementioned discrepancies between the compositional features of participating schools and those of secondary schools across England (see *Results*). However, at least some of these differences are attributable to the fact that the study sample included 31 schools for whose categorisation (e.g., independent, pupil referral unit, special school) national data are not available by phase of education (e.g., primary versus secondary); hence, national averages reported in the current study are for mainstream, non-independent secondary schools. Non-mainstream and independent schools are typically smaller, and non-mainstream schools contain a significantly higher proportion of pupils with SEN. Hence, when focusing on the 134 mainstream, non-independent schools in the study sample, size (study average: 1031 pupils per school; national average 1027.2) and SEN (study average: 16.1%; national average 14.1%) are much more comparable to the national picture.

Sixth, despite enabling more granular analyses of identities than has previously been possible, the underpinning dataset used in the current study did not contain information on gender and sexuality *expression*. The latter could feasibly mediate the relationship between gender and sexuality identities and wellbeing (e.g., ‘masculine girls’ and ‘feminine boys’ are likely targets for marginalization and discrimination, irrespective of their gender identity; conversely, an adolescent may identify as (for example) gay, but not disclose or express their sexual identity publicly). Future research focusing on both identities *and* expression could therefore significantly enhance our understanding of this important issue.

Finally, it should be acknowledged that there are other valid ways of conceptualising and coding data relating to transgender participants that differ from our approach (gender modality) [33, 34]. One option – as opposed to contrasting gender identity to administrative data on male/female assignment – is to ask participants to self-report if they consider themselves to be transgender (and indeed, such an item is being included in future iterations of the #BeeWell survey). A further issue regards the granularity of our groups and the fact that our approach to coding the data to create these groups may hide further wellbeing inequalities within these aggregated groups –an issue that should be examined in future research using these data. In this study, it would have been technically possible to disaggregate our data further (i.e., trans boys, trans girls, cis boys, cis girls, as opposed to simply transgender, cisgender) in order to identify potential further wellbeing inequalities hidden within our aggregated groups. For example, compared to cisgender boys, transgender boys likely experience a number of stressors and unique experiences related to their gender experience in a world that privileges and champions cisgender and heterosexual norms [78]; hence, conflating them (as in Model A) may mask important differences between these groups. Relatedly, we acknowledge the oppression towards cisgender (and transgender) girls in a patriarchal society, which means that aggregating cis girls and cis boys (as in Model B) may be similarly problematic. However, we opted not to undertake further disaggregation in view of both the statistical power implications (i.e., considerably reduced power, potentially leading to spurious results) and the fact that to do so would be ‘othering’ (i.e. it could be taken to imply that a trans boy’s identification as a boy is not the same as a cis boy’s identification as a boy).

## Conclusion

The analyses outlined in the current paper demonstrate substantial inequalities pertaining to gender and sexual identity across a range of wellbeing frameworks and domains in early adolescence, particularly for those adolescents whose identities transcend traditional gender identity binaries or who identify as gay/lesbian or bi/pansexual. To build on these findings, future research should routinely assess both gender *and* sexual identity in ways that enable adolescents to report diverse identities. An intersectional approach to examine how such inequalities vary across different socio-demographic characteristics (e.g., ethnicity) would also be welcome. There is a need for longitudinal research that can enable analyses focusing on the development of wellbeing inequalities, and the factors theorised to underpin them (e.g., experiences of discrimination and other mechanisms espoused in the minority stress model). In the interim, the evidence presented here is sufficient to warrant an urgent call for the prioritisation of improved prevention and intervention efforts that can better meet the needs of gender diverse and sexual minority youth.

### Electronic supplementary material

Below is the link to the electronic supplementary material.

Supplementary Material 1

## Data Availability

An anonymised version of the #BeeWell survey responses will be made publicly available in 2026. Due to ethical constraints this cannot be brought forward since participants have been given the right to withdraw their data until this point necessitating the need to maintain a securely stored pseudonymised version until this point. In addition, the non-self report data such as sex and free school meal eligibility will never be shared publicly due to the agreement in place with the local authorities who provided it. To request access to the #BeeWell data, please, contact Prof. Neil Humphrey at neil.humphrey@manchester.ac.uk.
