# Supplementary material for: Gender and sexual identity-based inequalities in adolescent wellbeing: findings from the #BeeWell Study
Source: BMC Public Health. 2023 Nov 9;23:2211. doi: 10.1186/s12889-023-16992-y (PMC10636841; doi:10.1186/s12889-023-16992-y)
Supplement: Supplementary file 1 — Supplementary Material 1 [file 12889_2023_16992_MOESM1_ESM.docx]

**Appendix 1**

**Part 1. Measurement model**

We compared the unidimensional, bifactor, and correlated factors structures for the three wellbeing frameworks, which enabled us to assess whether structural paths could be included to single hedonic/eudaimonic/complete state factors or whether these needed to be estimated at the domain level (e.g., negative affect). The bifactor model was included in case method factors (i.e., the fact that multiple scales were used) affected the fit of a unidimensional model. The bifactor model therefore allowed assessment of “essential unidimensionality” [58].

Fit indices are typically biased in favour of bifactor models [56], and the presence of multidimensionality in these models can lead to parameter bias [57] and problems for interpretability [55]. Accordingly, we did not judge the success of the bifactor model relative to the others on fit. Instead, we used two indices to gain insight into whether the general factor could be treated as essentially unidimensional, and therefore be used in structural models. Explained common variance (ECV) provides the percentage of variance that can be attributed to the general factor, while the percentage of uncontaminated correlations (PUC) reflects the proportion of correlations that inform only on the general factor (e.g., between items from different specific factors [57]). PUC moderates ECV such that at very high levels of PUC (>.80) lower ECV values can still reflect a strong general factor [57, 58]. In the absence of PUC > .80, ECV > .60 may be enough for bias to be slight.

Table A1.1 presents the relevant indices corresponding to each of the measurement models. Despite fit statistics generally favouring the bifactor solution, the PUC and ECV values in the hedonic, eudaimonic and complete state models are below the recommended thresholds noted above. This renders a bifactor model uninterpretable [55]. We therefore selected the correlated factors models since fit was acceptable and allowed consideration of pathways at the domain level. Accordingly, the structural models examined include structural paths from gender identity, sexual identity and co-variates to the domain level (e.g., negative affect) in each model, rather than, for instance, to general hedonic wellbeing.

**Table A1.1**

*Model Fit and Relevant Indices to Assess Model Structure.*

|  |  |  | Model fit indices | | | | | Model structure indices | |
| --- | --- | --- | --- | --- | --- | --- | --- | --- | --- |
| Model | Scales | Type | x2 | d.f. | RMSEA | CFI | TLI | PUC | ECV |
| Hedonic | Life satisfaction, positive affect, negative affect | Bifactor | 3713 | 34 | 0.055 | 0.963 | 0.940 | 0.636 | 0.620 |
|  |  | Correlated | 8516 | 42 | 0.074 | 0.915 | 0.888 |  |  |
|  |  | Unidimensional | 26066 | 44 | 0.172 | 0.737 | 0.672 |  |  |
| Eudaimonic | Autonomy, optimism, self-esteem, positive relationships | Bifactor | 8436 | 133 | 0.041 | 0.964 | 0.954 | 0.784 | 0.559 |
|  |  | Correlated | 15327 | 146 | 0.053 | 0.934 | 0.923 |  |  |
|  |  | Unidimensional | 73583 | 152 | 0.114 | 0.682 | 0.643 |  |  |
| Complete State Model of Mental Health | Mental wellbeing, symptoms of distress | Bifactor | 8397 | 102 | 0.047 | 0.964 | 0.952 | 0.515 | 0.720 |
|  |  | Correlated | 17857 | 118 | 0.059 | 0.935 | 0.926 |  |  |
|  |  | Unidimensional | 38354 | 119 | 0.094 | 0.833 | 0.809 |  |  |

**Part 2. Sensitivity analysis**

**Table A1.2.**

*SEM estimates for gender identity, sexual identity, and covariates (hedonic model)*

|  | Positive Affect | | | | | | Negative Affect | | | | | | Life satisfaction | | | | | |
| --- | --- | --- | --- | --- | --- | --- | --- | --- | --- | --- | --- | --- | --- | --- | --- | --- | --- | --- |
|  | Model A | | | Model B | | | Model A | | | Model B | | | Model A | | | Model B | | |
|  | B | | S.E. | B | | S.E. | B | | S.E. | B | | S.E. | B | | S.E. | B | | S.E. |
| Year 10 (ref: year 8) | -0.23 | *** | 0.01 | -0.23 | *** | 0.01 | 0.09 | *** | 0.01 | 0.10 | *** | 0.02 | -0.24 | *** | 0.01 | -0.24 | *** | 0.01 |
| FSM (ref: no FSM) | -0.11 | *** | 0.01 | -0.11 | *** | 0.01 | -0.01 |  | 0.01 | -0.01 |  | 0.01 | -0.13 | *** | 0.01 | -0.13 | *** | 0.01 |
| SEN (ref: no SEN) | -0.04 | * | 0.02 | 0.00 |  | 0.02 | 0.04 | * | 0.02 | -0.05 | ** | 0.02 | 0.00 |  | 0.02 | 0.05 | ** | 0.02 |
| Black ethnicity (ref: white) | 0.12 | *** | 0.02 | 0.11 | *** | 0.02 | -0.16 | *** | 0.03 | -0.14 | *** | 0.03 | 0.01 |  | 0.02 | -0.01 |  | 0.02 |
| Asian ethnicity (ref: white) | 0.06 | *** | 0.02 | 0.06 | ** | 0.02 | -0.21 | *** | 0.02 | -0.20 | *** | 0.03 | 0.04 | ** | 0.02 | 0.04 | ** | 0.02 |
| Chinese ethnicity (ref: white) | -0.06 |  | 0.06 | -0.07 |  | 0.05 | 0.06 |  | 0.06 | 0.12 | * | 0.06 | -0.15 | * | 0.07 | -0.17 | ** | 0.06 |
| Other ethnicity (ref: white) | 0.06 |  | 0.03 | 0.06 |  | 0.04 | -0.12 | ** | 0.04 | -0.13 | ** | 0.05 | 0.00 |  | 0.03 | 0.01 |  | 0.04 |
| Mixed ethnicity (ref: white) | 0.03 |  | 0.02 | 0.03 |  | 0.03 | -0.07 | ** | 0.03 | -0.06 | * | 0.03 | -0.05 | * | 0.02 | -0.06 | ** | 0.02 |
| Unclassified ethnicity (ref: white) | 0.00 |  | 0.04 | 0.00 |  | 0.04 | -0.14 | ** | 0.05 | -0.13 | ** | 0.05 | -0.05 |  | 0.04 | -0.05 |  | 0.04 |
| Girl (incl. trans) (ref: Boy (incl. trans)) | -0.28 | *** | 0.01 |  |  |  | 0.57 | *** | 0.01 |  |  |  | -0.33 | *** | 0.01 |  |  |  |
| Non-binary gender identity (ref: Boy (incl.trans)) | -0.43 | *** | 0.04 |  |  |  | 0.61 | *** | 0.04 |  |  |  | -0.53 | *** | 0.03 |  |  |  |
| Other gender identity (ref: Boy (incl.trans)) | -0.35 | *** | 0.03 |  |  |  | 0.54 | *** | 0.03 |  |  |  | -0.43 | *** | 0.03 |  |  |  |
| Prefer not to say gender identity (ref: male (incl.trans)) | -0.20 | *** | 0.03 |  |  |  | 0.25 | *** | 0.03 |  |  |  | -0.23 | *** | 0.02 |  |  |  |
| Bisexual/Pansexual (ref: heterosexual) | -0.51 | *** | 0.02 | -0.56 | *** | 0.02 | 0.81 | *** | 0.02 | 0.91 | *** | 0.02 | -0.62 | *** | 0.02 | -0.68 | *** | 0.02 |
| Gay/Lesbian (ref: heterosexual) | -0.54 | *** | 0.04 | -0.56 | *** | 0.04 | 0.80 | *** | 0.03 | 0.83 | *** | 0.04 | -0.65 | *** | 0.03 | -0.67 | *** | 0.03 |
| Prefer not to say sexual identity (ref: heterosexual) | -0.12 | *** | 0.02 | -0.12 | *** | 0.02 | 0.23 | *** | 0.02 | 0.23 | *** | 0.02 | -0.14 | *** | 0.02 | -0.15 | *** | 0.02 |
| Other sexual identity (ref: heterosexual) | -0.20 | *** | 0.03 | -0.20 | *** | 0.03 | 0.31 | *** | 0.03 | 0.31 | *** | 0.03 | -0.25 | *** | 0.03 | -0.24 | *** | 0.03 |
| Transgender (ref: cisgender) |  |  |  | -0.23 | *** | 0.02 |  |  |  | 0.28 | *** | 0.02 |  |  |  | -0.28 | *** | 0.02 |
| Standardized coefficients; *** p ≤ 0.001, ** p ≤ 0.01, * p ≤ 0.05 | | | |  |  |  |  |  |  |  |  |  |  |  |  |  |  |  |

**Table A1.3.**

*SEM estimates for gender identity, sexual identity, and covariates (complete state model)*

|  | Mental wellbeing | | | | | | Symptoms of distress | | | | | |
| --- | --- | --- | --- | --- | --- | --- | --- | --- | --- | --- | --- | --- |
|  | Model A | | | Model B | | | Model A | | | Model B | | |
|  | B | | S.E. | B | | S.E. | B | | S.E. | B | | S.E. |
| Year 10 (ref: year 8) | -0.16 | *** | 0.01 | -0.17 | *** | 0.01 | 0.06 | *** | 0.01 | 0.07 | *** | 0.02 |
| FSM (ref: no FSM) | -0.12 | *** | 0.01 | -0.11 | *** | 0.01 | 0.04 | *** | 0.01 | 0.04 | ** | 0.01 |
| SEN (ref: no SEN) | -0.08 | *** | 0.02 | -0.02 |  | 0.02 | 0.06 | *** | 0.02 | -0.03 | * | 0.02 |
| Black ethnicity (ref: white) | 0.14 | *** | 0.03 | 0.12 | *** | 0.03 | -0.17 | *** | 0.03 | -0.15 | *** | 0.03 |
| Asian ethnicity (ref: white) | 0.14 | *** | 0.02 | 0.13 | *** | 0.02 | -0.19 | *** | 0.02 | -0.18 | *** | 0.03 |
| Chinese ethnicity (ref: white) | -0.01 |  | 0.06 | -0.02 |  | 0.06 | 0.03 |  | 0.06 | 0.08 |  | 0.06 |
| Other ethnicity (ref: white) | 0.17 | *** | 0.03 | 0.18 | *** | 0.04 | -0.11 | ** | 0.04 | -0.12 | ** | 0.05 |
| Mixed ethnicity (ref: white) | 0.03 |  | 0.02 | 0.02 |  | 0.03 | -0.07 | ** | 0.03 | -0.05 | * | 0.03 |
| Unclassified ethnicity (ref: white) | 0.04 |  | 0.06 | 0.03 |  | 0.06 | -0.13 | ** | 0.04 | -0.13 | ** | 0.05 |
| Girl (incl. trans) (ref: Boy (incl. trans)) | -0.40 | *** | 0.01 |  |  |  | 0.58 | *** | 0.01 |  |  |  |
| Non-binary gender identity (ref: Boy (incl.trans)) | -0.58 | *** | 0.04 |  |  |  | 0.66 | *** | 0.04 |  |  |  |
| Other gender identity (ref: Boy (incl.trans)) | -0.48 | *** | 0.04 |  |  |  | 0.58 | *** | 0.03 |  |  |  |
| Prefer not to say gender identity (ref: male (incl.trans)) | -0.30 | *** | 0.03 |  |  |  | 0.28 | *** | 0.03 |  |  |  |
| Bisexual/Pansexual (ref: heterosexual) | -0.65 | *** | 0.02 | -0.72 | *** | 0.02 | 0.82 | *** | 0.02 | 0.93 | *** | 0.02 |
| Gay/Lesbian (ref: heterosexual) | -0.66 | *** | 0.03 | -0.67 | *** | 0.03 | 0.81 | *** | 0.03 | 0.85 | *** | 0.03 |
| Prefer not to say sexual identity (ref: heterosexual) | -0.21 | *** | 0.02 | -0.22 | *** | 0.02 | 0.23 | *** | 0.02 | 0.24 | *** | 0.02 |
| Other sexual identity (ref: heterosexual) | -0.30 | *** | 0.03 | -0.29 | *** | 0.03 | 0.34 | *** | 0.03 | 0.33 | *** | 0.03 |
| Transgender (ref: cisgender) |  |  |  | -0.32 | *** | 0.02 |  |  |  | 0.30 | *** | 0.02 |
| Standardized coefficients; *** p ≤ 0.001, ** p ≤ 0.01, * p ≤ 0.05 | | | |  |  |  |  |  |  |  |  |  |
